# Models that include supercoiling of topological domains reproduce several known features of interphase chromosomes

**DOI:** 10.1093/nar/gkt1353

**Published:** 2013-12-22

**Authors:** Fabrizio Benedetti, Julien Dorier, Yannis Burnier, Andrzej Stasiak

**Affiliations:** ^1^Center for Integrative Genomics, University of Lausanne, 1015 Lausanne, Switzerland, ^2^Vital-IT Group, SIB Swiss Institute of Bioinformatics, 1015 Lausanne, Switzerland and ^3^Laboratory of Particle Physics and Cosmology, Institute of Theoretical Physics, Ecole polytechnique fédérale de Lausanne EPFL, 1015 Lausanne, Switzerland

## Abstract

Understanding the structure of interphase chromosomes is essential to elucidate regulatory mechanisms of gene expression. During recent years, high-throughput DNA sequencing expanded the power of chromosome conformation capture (3C) methods that provide information about reciprocal spatial proximity of chromosomal loci. Since 2012, it is known that entire chromatin in interphase chromosomes is organized into regions with strongly increased frequency of internal contacts. These regions, with the average size of ∼1 Mb, were named topological domains. More recent studies demonstrated presence of unconstrained supercoiling in interphase chromosomes. Using Brownian dynamics simulations, we show here that by including supercoiling into models of topological domains one can reproduce and thus provide possible explanations of several experimentally observed characteristics of interphase chromosomes, such as their complex contact maps.

## INTRODUCTION

Several recent papers have shown that chromatin fibres in interphase chromosomes are organized into ∼1 Mb large domains that can be seen on 3C contact maps as sharply delimited regions with highly increased frequency of internal contacts (1–4). Although these domains, better known as topological domains, can be easily pinpointed on 3C contact maps, little is known about their structure and organization. It is known though that borders of topological domains are determined by combinations of specific DNA binding proteins including CTCF (2,4). Deposited data reveal that contacts between loci located in the same domain are 2- to 3-fold more frequent than between loci with the same genomic distance but located in neighbouring domains (2,3). In addition, the probability of interloci contacts decreases slower for distances of typical topological domains than for larger distances (2,3,5). The underlying mechanisms responsible for the increase of contacts within individual topological domains are not established yet. In one of the first 3C studies that demonstrated the existence of topological domains, the authors presented a schematic model of the organization of these domains (2). According to that model, chromatin stretches forming individual topological domain fold into segregated globules. Once such globules are formed and maintained, the contacts between fluctuating segments of the same globule are expected to be much more frequent than contacts between segments belonging to two neighbouring, but segregated, domains. It was not explained though what could lead to the formation of such globular arrangements (2). In a more recent modelling study, Barbieri *et al.* (6) proposed that separate globules can form by interaction with polyvalent binders that only bind within a given topological domain. Although that model can reproduce experimental 3C data, it would require >2000 kinds of binders to explain all separate topological domains in human genome. Barbieri *et al.* (6) did not propose though what could be these specific binders.

We investigate here whether a simpler model, not necessitating large number of different binders, can qualitatively and quantitatively reproduce experimental 3C data obtained in recent studies of interphase chromosomes in eukaryotic cells (2,3). Our model is designed to reflect the situation where unconstrained supercoiling acts on chromatin fibres that are sparsely attached at specific sites to nuclear granules. Each such attachment locally limits the possibility of axial rotation of chromatin fibres. Our model is supported by recent reports indicating that boundary elements of topological domains are attached to nuclear granules (7), and recent reports indicating that chromatin fibres are supercoiled (8). We further assume that boundary elements of the same topological domains usually bind to different nuclear granules that can slowly move within the nucleus. Such a situation would lead then to formation of localized supercoiled domains without actual closures of individual topological domains into loops. Unfortunately, simulation of such a system would be complex and require many arbitrary assumptions regarding, for example, diffusion coefficients of various nuclear granules. To simplify the simulation, and have the modelled topological domains free to take the structure dictated by supercoiling, we closed modelled topological domains with accessory linker chains (Supplementary Figures S1 and S2). Such linkers do not force the two ends of a given topological domains to stay together but rather let them to fluctuate around positions dictated by supercoiling of modelled topological domains. A similar behaviour would be expected for supercoiled topological domains where boundary elements are attached to different nuclear granules. Importantly, the linker chains serve only an accessory role and are not entered into the statistics of contacts. Using the model described earlier in the text, we checked whether supercoiling can cause formation of topological domains, i.e. regions with 2- to 3-fold increased frequency of contacts as compared with loci with similar genomic distance but located in different topological domains (2). We also test whether supercoiling can reproduce the experimentally observed α exponent, which characterizes how quickly the probability of contacts between chromosomal regions decreases with their genomic distance.

## MATERIALS AND METHODS

Smaller scale simulations of chromosome fragments with 2 or 3 topological domains were performed using a model where diameter of beads corresponded to the diameter of 30-nm chromatin fibres. Beads of this size represent then 4000 bp each (9). The modelled topological domains ranged from 100 to 200 beads (see Supplementary Figure S3). The larger loops thus correspond to ∼800 000 bp and are close to the average size of topological domains (2,5). The extent of introduced supercoiling (10,11) ranged from ΔLk = −1 to −8 per 100 beads for weakly and strongly supercoiled topological domains, respectively. As a control, we also performed simulations, where individual topological domains were modelled as simple non-supercoiled loops in which we imposed contacts between border elements belonging to the same topological domain. Large-scale simulations of chromosome fragments containing 50 topological domains were performed using a coarser graining approach where one bead corresponded to 25 000 bp. To induce formation of interwound supercoils in individual topological domains, we maintained the level of supercoiling corresponding to ΔLk ≈ −8/100 beads. For contact maps obtained in small-scale simulations, we considered two beads in a contact when their centre-to-centre distance was <6 beads’ diameters. This contact radius was set to account for sizes of transcription factories that range up to 180 nm (12) and polycomb bodies that exceed 200 nm (13). Transcription factories and polycomb bodies mediate contacts between transcriptionally active and inactive chromatin regions, respectively (5,7,14,15). These contacts are detected then by high-throughput 3C methods. In large-scale simulations, we considered two beads in contacts when their centre-to-centre distance exceeded the size of two beads. To be able to maintain torsional stress in individual topological domains, we have closed them with accessory chains that behaved in the same way as the rest of the chains. These chains were not considered though in the contact matrices or in the statistics needed to calculate the α exponent. To mimic the effect of high concentration of chromatin in eukaryotic nuclei, we performed the simulations setting the concentration to 20% volume occupation (16). The data were collected for simulations performed under periodic boundary conditions. For more detail see Supplementary Data.

## RESULTS

We modelled thermally fluctuating chromatin fibres as worm-like beaded chains with bending and torsional resistance using HooMD-blue program, http://codeblue.umich.edu/hoomd-blue (17,18). According to recent proposals, we assumed that borders of topological domains are firmly attached to nuclear granules such as transcription factories or polycomb bodies (7). We further assumed that this attachment limits the possibility of axial rotation of chromatin fibres, although the anchoring nuclear granules are essentially free to move within the nucleus. Knowing that transcription induces supercoiling (19–21) and considering recent reports revealing the presence of various levels of unconstrained supercoiling in chromatin (8,22), we modelled chromatin stretches between two consecutive attachment points as strongly or weakly supercoiled. Strongly supercoiled stretches were taking the form of interwound superhelices (23,24), whereas the extent of torsional stress in weakly supercoiled domains was not sufficient to induce their interwinding. To account for high crowding of chromatin fibres inside nuclei, we performed simulations under conditions where simulated chains occupy 20% of the available volume (16). Further details of the simulation procedure are described in ‘Materials and Methods’ section and in Supplementary Data.

### Effect of supercoiling on the contact maps

**Figure 1.**
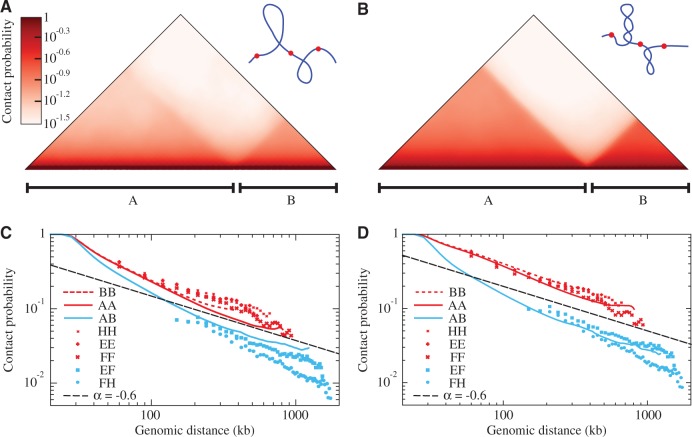
Models that impose strong supercoiling of individual topological domains recapitulate experimental 3C data. (**A**, **B**) Contact maps obtained for simulated chromatin fibres composed of two topological domains A and B with sizes corresponding to 800 and 400 kb that were weakly (panel A) or strongly supercoiled (panel B) (with ΔLk of −1 or of −8 per 400 kb, respectively). The two drawings schematically present the two systems. (**C**, **D**) Comparison of the average contact probability profiles for loci located in the same or neighbouring topological domains for modelled chromatin fibres composed of weakly or strongly supercoiled topological domains (panels C and D, respectively) with experimental 3C data. Notations AA, BB and AB indicate intra- and interdomain contacts, respectively, for simulated topological domains. The experimental 3C data points (shown as scatter plots in C–D) correspond to contacts within and between topological domains E, F and H presented in Figure 1 of Nora *et al.* (3). The dashed line indicates the slope corresponding to the α exponent of −0.6.

To evaluate the effect of different levels of supercoiling in models aiming to recapitulate experimentally determined 3C data, we compared results of simulations of chromatin fragments composed of two topological domains with the size of 800 and 400 kb, which were supercoiled to a low and high level. We first looked at contact maps. As shown in Figure 1A and B, an increase of supercoiling accentuated characteristic triangles visible on contact maps of simulated chromatin fragments. In fact, such accentuated triangles visible in the experimental contact maps have led to the discovery of topological domains in interphase chromosomes (2).

To evaluate the effect of supercoiling in a more quantitative way, we compared how the average contact probability decreases with the separating genomic distance in simulations and in experiments. As experimental data, we took first intra- and inter-domain contact probabilities involving topological domains E, F and H in the X-chromosome inactivation centre of mouse embryonic stem cells that were presented in Figure 1 of Nora *et al.* (3). As shown in Figure 1C and D, both models closely reproduced the experimentally determined α exponent for contacts occurring within and between neighbouring topological domains E, F and H. However, only the model with strong supercoiling recapitulated the ratio of contacts occurring within individual topological domains and between neighbouring topological domains in these undifferentiated embryonic stem cells (3). It is important to mention though, that a model with a weak supercoiling matched better experimental data obtained by Nora *et al.* (3) for the same genomic region but in differentiated mouse embryonic fibroblasts cell lines (see Supplementary Figure S6A and B). This later result agrees with the notion that supercoiling in topological domains is dynamic and may change with cell activity (8,22). Interestingly, experimental data obtained by Nora *et al.* for the same genomic region but in mutant cells defective in H3K27me3 histone methylation showed that interdomain contacts were decaying significantly quicker than in our two models (see Supplementary Figure S5C and D).

### Simple loops fail to reproduce 3C data

**Figure 2.**
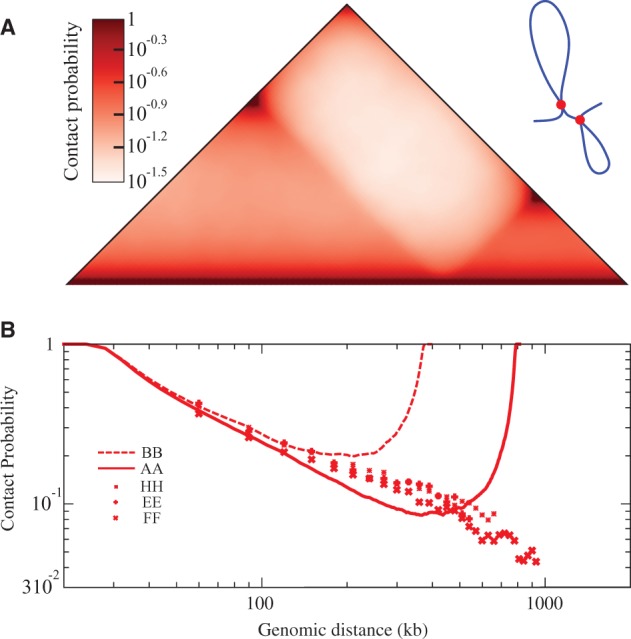
Simple loop models of topological domains do not recapitulate the 3C data. (**A**) Contact maps obtained for a simple loop model of two topological domains with sizes corresponding to 800 and 400 kb, respectively. (**B**) Contact probability profiles obtained in simulations of topological domains as non-supercoiled loops (indicated with continuous lines) and 3C data for individual topological domains E, F and H presented in Figure 1 of Nora *et al.* (3). Notice that in contrast to the model presented in Figure 1, the simple loop models fail to reproduce the experimental data.

The results presented earlier in the text were obtained for a model that does not impose contacts between boundary elements of topological domains but permits the domains to take the structure dictated by the supercoiling of modelled chromatin portions between the boundary elements. In the absence of supercoiling and without the accessory linker chains such a model would behave as a simple generic polymer and would show no increased contact frequency within individual topological domains as long as the ends of topological domains were attached to independent granules that are free to move within the nucleus. However, if ends of individual topological domains were attaching to the same nuclear granules then they could form simple loops that are likely to result in a local increase of contacts within individual topological domains even without supercoiling. Therefore, we also modelled such systems to see whether they can recapitulate the experimental 3C data. Figure 2 presents the results of simulations analysing contact maps and the α exponent values of a system composed of two simple loops formed by topological domains of the same size as these analysed in Figure 1. As we can see, the presence of simple loops results in formation of sharply defined triangles in the contact map. These triangles, however, show high intensity of contacts around their vertices that are distal from the diagonal (Figure 2A). These contacts are natural consequence of the fact that in this particular model the border elements belonging to the same topological domains were brought together. This feature is seen even more clearly in profiles of α exponent values obtained for loop models of topological domains (see Figure 2B). Within individual loops one initially observes that the probability of contacts decreases with the separating genomic distance (See Figure 2B). However, as this distance becomes larger than the half of the loop, one observes that the contact frequency increases and finally reaches the value typical for small genomic separations. It is important to stress here that neither experimental 3C data (see Figure 2B) nor our model, in which we did not force border elements of the same topological domains to interact with each other, shows such features (see Figure 1). Simple loop models can be modified to avoid that regions located close to two border elements show such high probability of contacts. For example, it is possible that simple loops form only for a fraction of time and stay open for the rest. Such a modification decreases to some extent the rising portion of the α exponent profile for distances larger than half of the loop. However, it also decreases the α exponent values for distances smaller than half of the loop, resulting in the α exponent that is not consistent with the experimental data. It should be mentioned here that also in simulations of supercoiled simple loops one observes the ‘U’ shape of the contact probability profiles (data not shown). This result is the consequence of the fact that regions close to two border elements of the same domain are brought together by the loop closure.

### Supercoiling compactifies individual topological domains

**Figure 3.**
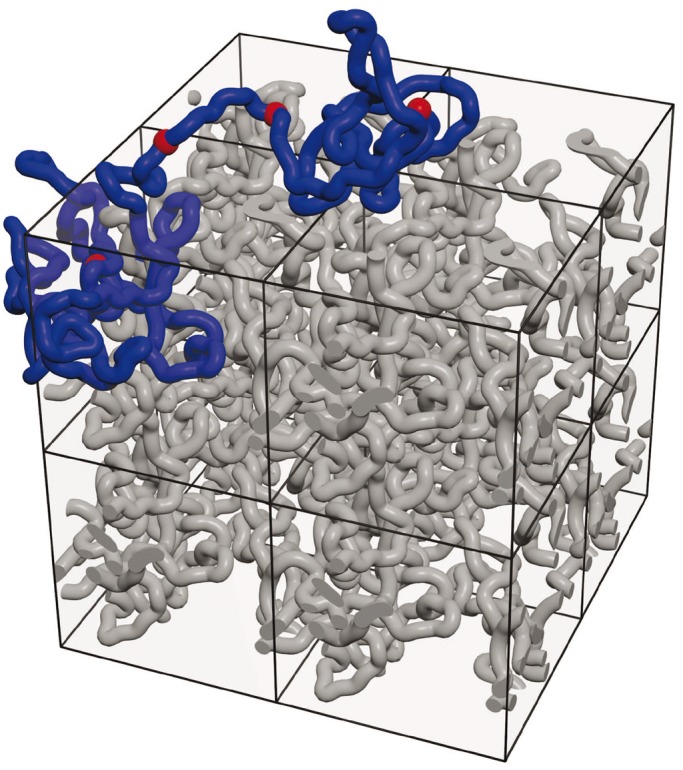
Simulations reveal local compaction of individual topological domains. Snapshot from the simulation run that provided the data for Figure 1B. One chromatin fragment consisting of two supercoiled topological domains with sizes corresponding to 800 and 400 kb was simulated under periodic boundary conditions. For better visibility, one of the periodic copies of the simulated fragment composed of two supercoiled topological domains is partially ‘extracted’ from highly crowded melt composed of other periodic copies of the same molecule. The image shows eight periodic copies of the actual simulation box. Notice local compaction of individual supercoiled topological domains.

After demonstrating that simple closed loop models of topological domains are inconsistent with the experimental 3C data (see Figure 2), we concentrate on further characterization of our model presented in Figure 1. Although contact maps and α exponent profiles provide quantifiable characteristics of overall shapes adopted by the topological domains simulated by us, it is also important to be able to see the simulated configurations. Figure 3 shows a snapshot from the simulation run investigating the shape of a fragment composed of two strongly supercoiled topological domains with sizes corresponding to 800 and 400 kb, respectively. The snapshot is from the simulation run that produced contact map presented in Figure 1B. The simulation was performed under periodic boundary conditions, which is the method of choice to simulate highly concentrated polymer chains. Simulated polymer molecules evolve during simulations like if they were surrounded by other polymer molecules, although the other molecules are periodic copies of the same molecule, and this makes the simulation process much more efficient (25). In our case, we simulated one chromatin fragment composed of two topological domains. The size of the cubic simulation box was adjusted in such ways that one simulated chromatin fragment occupies 20% of its volume. For the purpose of visualization, one of the periodic copies of the simulated chromatin fragment composed of two topological domains is partially extracted from the ‘melt’ constituted by the other periodic copies. Notice that individual topological domains are locally compacted. This explains why contacts within individual topological domains are more frequent than between neighbouring domains. Because models implicating strong supercoiling of topological domains showed a better agreement with the majority of experimental 3C data obtained for wild-type cells (see Figure 1), in further work we only considered models with strongly supercoiled topological domains.

### Complex contact maps can be caused by transient border elements

**Figure 4.**
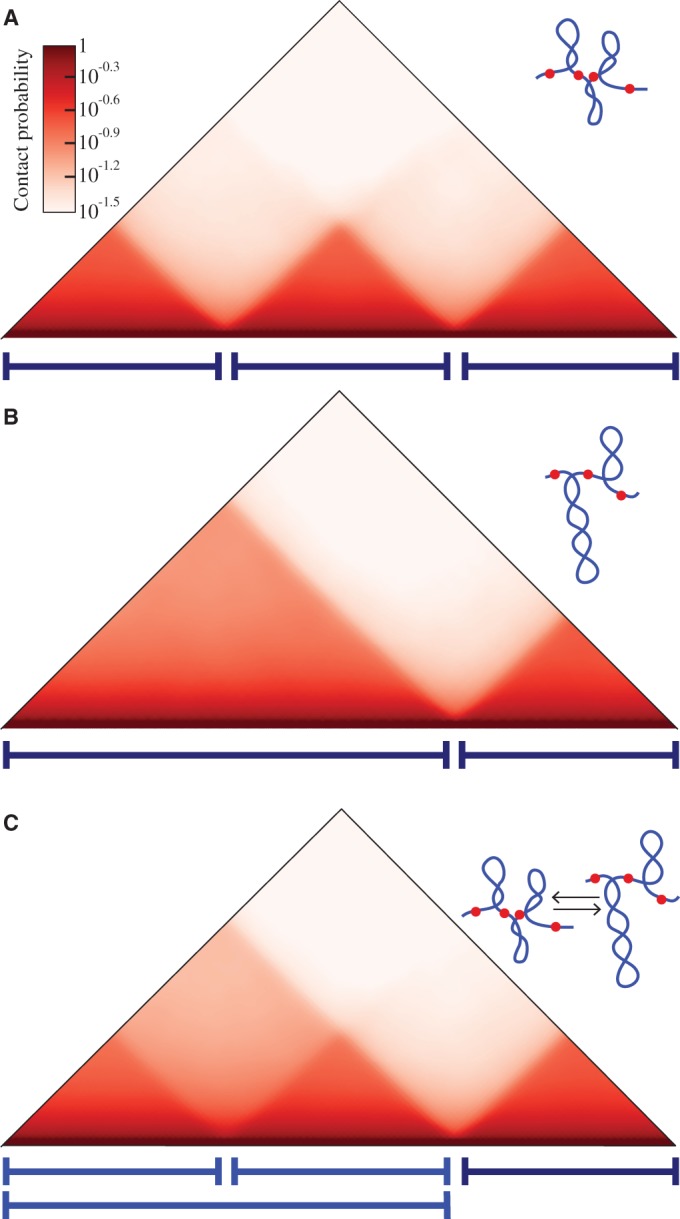
Transient border elements explain the substructure of contact maps. (**A**, **B**) Contact maps obtained after simulations of a chromosome region that in the first case (A) forms three supercoiled topological domains (where the first one is roughly twice bigger than the second) and in the second case (B) forms two supercoiled domains, as one transient border element is active in this case. (**C**) Combined contact matrix corresponding to the situation where the transient border element is active 50% of the time. Schematic drawings present situations corresponding to respective contact maps.

High resolution contact maps obtained by Dixon *et al.* (2) and by Nora *et al.* (3) reveal that although some topological domains show a smooth gradation pattern of intradomain contacts and their contact maps look like nice triangles [e.g. the topological domain shown on the right of Figure 2E in (2)], the majority of domains show more complex contact maps. These more complex contact maps look like superpositions of semitransparent triangles of various sizes [see Figures 1A and 2E in (2)]. Probably the simplest interpretation of such a complex contact map is that the division of a given chromosome region into sequential topological domains was not the same in every cell at the moment when the cells were fixed for the 3C analysis. The experimentally obtained contact maps suggest that some boundary elements of topological domains behaved as transient. To test this interpretation, we modelled a situation where a given chromosomal region has three permanent and one transient boundary element. Figure 4 shows contacts maps obtained for two different states of this system and also the averaged contact map, assuming that the transient boundary element is active 50% of the time. The averaged contact map shows the characteristic substructure similar to experimental contact maps of several chromosomal regions analysed by Dixon *et al.* (2). The example of the superposition shown in Figure 4C is relatively simple and does not explain all contact maps observed experimentally. However, all experimental contact maps are likely to be explained by the presence of several transient boundary elements with varying duration times.

### Experimentally observed transition between two regimes of α exponent is recapitulated by our model

**Figure 5.**
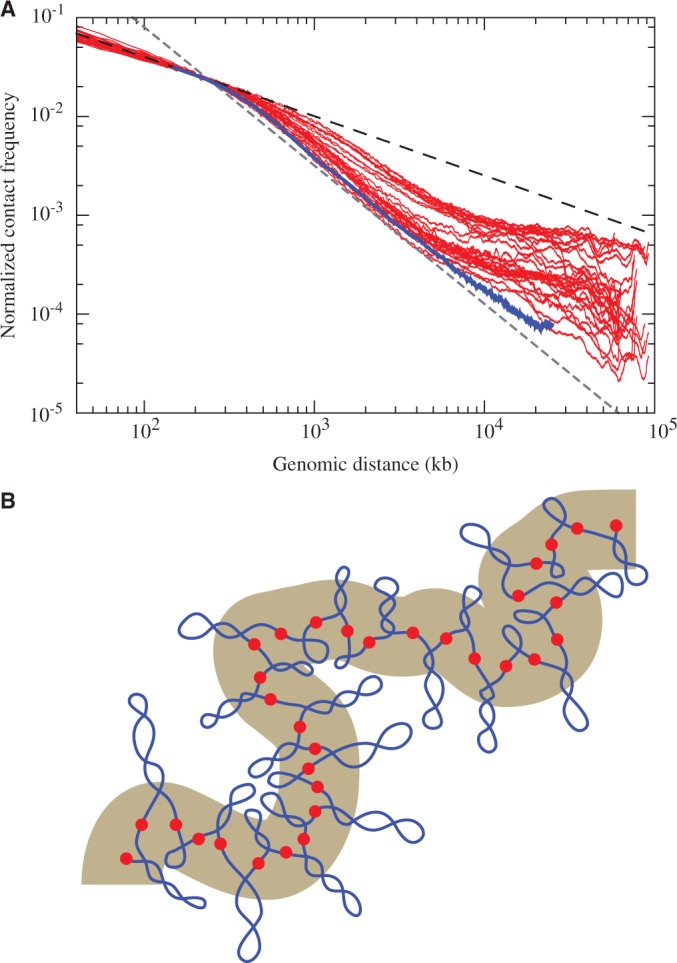
Model of a large chromosome fragment composed of supercoiled topological domains recapitulates the experimentally observed changes of α exponent as the genomic separation between contacting regions increases. (**A**) The contact decay profile obtained in simulations of a large chromosome fragment with ∼60 Mb (blue line) is overlaid on experimentally determined contact decay profiles extracted from 3C data collected by Dixon *et al.* (2). The experimental profiles are for several different chromosomes with the size of ∼60 Mb, which were analysed in different cell lines. Each red line is for a given chromosome and a given cell line. Experimental profiles were rescaled so that the contact frequency observed at loci separation of 200 kb corresponded to contact probability observed in simulations for separations corresponding to 200 kb. The simulated profile starts with the size of five beads, as below that number the coarse graining is not suited to adequately evaluate contact frequencies. The simulated profile is shown up to 25 Mb, as beyond that scale the values were strongly affected by insufficient simulation time. The thickness of the blue line is larger than one standard deviation error bars evaluated using the blocking method (27). The dashed black and grey lines indicate slopes corresponding to α exponents of −0.6 and −1.4, respectively. (B) Schematic drawing of a large chromosome fragment composed of supercoiled domains with different sizes and different extent of supercoiling. On a large scale such fragment is expected to behave like an elastic generic polymer (shown as a thick grey tube).

Simulation results presented up to now were obtained using a model where the diameter of beads corresponds to the diameter of 30-nm chromatin fibres. Taking the linear density of 30-nm chromatin fibres (9), one can calculate that one bead represents a chromatin fragment with ∼4000 bp. Using such a model, it is relatively easy to simulate short chromosome fragments of up to 2 Mb composed of two or three topological domains and requiring ∼500 beads. However, entire human chromosomes are much larger and have the average size of ∼150 Mb. Modelling them with beads corresponding to 4000 bp each would require unrealistic computation times especially for simulations of highly crowded systems composed of supercoiled domains. A coarse graining approach is needed in such a case. Importantly, using coarse graining, one can still adequately model large-scale properties such as the α exponent over distances spanning entire chromosomes. Therefore, we decided to model a large chromosome fragment with ∼60 Mb [size range of short human chromosomes or individual arms of long human chromosomes (26)] using a model where each bead represents 25 000 bp. Our modelled fragment was composed of 50 topological domains, with different sizes that were randomly sampled from the distribution of topological domain’s sizes determined experimentally (2) (see Supplementary Figure S6). It is important to mention here that the scale of our large chromosome fragment model is effectively set by the average number of beads used to model individual topological domains, which in reality have the average size of ∼1 Mb. Figure 5A shows that contact probability profile of simulated 60 Mb fragment closely resembles contact probability profiles that can be extracted from the data deposited by Dixon *et al.* (2). To have a more meaningful comparison, we only show experimental data obtained for different chromosomes having the size of ∼60 Mb and which were analysed in several different cell lines (2). It is visible that for genomic separations smaller than the size of topological domains, the α exponent has a value of about −0.6 both for modelled and experimental data. At larger genomic separations (>0.5 Mb), the α exponent tends to be approximately −1.4 for our modelled fragment with 50 topological domains (see Figure 5A). A similar tendency is observed in experimentally determined contact decay profiles for several chromosomes with ∼60 Mb that were studied in various cell lines by Dixon *et al.* (2) (see Figure 5A). However, experimentally determined contact decay profiles showed α exponent ranging from −1 to about −1.5 (see experimental profiles shown in Figure 5A). It is important to add here that despite over two months of equilibration using a fast graphic card, our modelled chromosome fragment with 50 supercoiled topological domains was not yet equilibrated and the α exponent was shifting with the time towards more negative values. However, as pointed out by Rosa and Everaers (28) also, real chromosomes are unlikely to have the time to equilibrate in actively growing cells. Schematic drawing in Figure 5B schematically presents large chromosome fragments composed of sequentially placed supercoiled loops. Such a fragment, despite being composed of loops, is expected to behave on a large scale like a generic flexible polymer chain. Such chains at high concentration are expected to show at equilibrium the α exponent of −1.5 (29,30). However, the time needed for a chromosome to reach the equilibrium after it started to decondense in the interphase is presumably much longer than the average time of duration of interphase (28). If that is the case, chromosomes do not have time to equilibrate and will therefore form so-called fractal globules characterized by the α exponent of −1, as observed and interpreted by Lieberman-Eiden *et al.* (5,29). With longer equilibration time, fractal globule is expected to progressively change towards the equilibrium globule with the α exponent progressively changing from −1 to −1.5 (5,29).

## DISCUSSION

We have presented a relatively simple model of organization of topological domains that agrees with the available 3C data. It is important though to discuss how our model compares with other models in the literature. Because the discovery of topological domains in eukaryotic chromosomes is relatively recent, only few papers discussed their possible structure (2,31), and we are aware of only one modelling study directly addressing the structure of topological domains (6). The model proposed by Barbieri *et al.* involved specific binders that only aggregate together chromatin portions belonging to the same topological domain. That model is able to imitate general characteristics of topological domains, but it requires that each topological domain should have domain-specific binders recognizing it. Because there are several thousands of topological domains in genomes of higher eukaryotes, one would also need to have several thousand species of these specific binders. In addition, each topological domain would need to have highly specific markers enabling it to attract its specific binders. Barbieri *et al.* did not propose, what could be these specific markers and specific binders for each topological domain.

Several earlier papers, preceding the discovery of topological domains, proposed models where chromosomes are organized into sequentially arranged closed loops (32,33). We showed, however (see Figure 2), that if topological domains were forming simple loops, they would be unlikely to reproduce the experimental 3C data. The problem with simple loops is that the probability of contacts between sites located in the same loop reaches the smallest value for the genomic distance corresponding to the half size of a given loop and then increases again. Such behaviour is not observed in the analysed 3C data (3). In the model that we propose here, border elements of a given topological domains are not enforced to be in a contact. They are essentially free to move independently from each other. However, it is known that supercoiling causes formation of interwound plectonemes, as this permits to decrease the elastic energy of torsionally stressed elastic filaments such as DNA (34,35) or protein–DNA complexes (36). As a consequence, the average distance between border elements of the same topological domains and also between any two sites in the same topological domain is on average closer to each other as compared with the situation where topological domain were not supercoiled and thus behaved as generic polymer chains. This local compactification of topological domains explains then why the contact frequency between sites located in the same topological domain is higher than what would be expected for a generic polymer model. It is important to add here that our model of topological domains reproduced not only the typical triangular pattern on the contact maps but also the two regimes of the α exponent characterizing chromatin organization at the scale below and above 1 MB (1–5,29). Because the average size of topological domains is of ∼1 MB (2,3), the α exponent values for distances <1 MB are reflecting mainly the frequency of contacts within individual topological domains. However, for distances >1 MB, the interacting sites belong to different topological domains. Our proposal that an increased frequency of intradomain contacts is due to supercoiling agrees with two recent studies that detected unconstrained transcription-induced supercoiling in topological domains of interphase chromosomes (8,22).

When this article was under review, a new study was published that combined high resolution 3C data and polymer modelling to elucidate the structure of bacterial chromosomes (37). It was revealed that bacterial chromosomes are composed of regions with locally increased frequency of contacts that behave in an analogous way to topological domains in eukaryotic chromosomes (2). Modelling studies performed by Le *et al.* (37) suggested that individual topological domains are composed of supercoiled regions forming plectonemes and that the border between topological domains are plectoneme-free.

## SUPPLEMENTARY DATA

Supplementary Data are available at NAR Online.

## FUNDING

Swiss National Science Foundation grant [31003A_138267 to A.S.]. Funding for open access charge: Waived by Oxford University Press. 

*Conflict of interest statement*. None declared.

## Supplementary Material

Supplementary Data
