# Supplementary material for: Models that include supercoiling of topological domains reproduce several known features of interphase chromosomes
Source: Nucleic Acids Res. 2013 Dec 22;42(5):2848–55. doi: 10.1093/nar/gkt1353 (PMC3950722; doi:10.1093/nar/gkt1353)
Supplement: Supplementary Data [file supp_42_5_2848__index.html]

Models that include supercoiling of topological domains reproduce several known features of interphase chromosomes — Supplementary Data 

# Models that include supercoiling of topological domains reproduce several known features of interphase chromosomes

## Supplementary Data

files

**Files in this Data Supplement:**

- Supplementary Data - docx file
