# Supplementary material for: Models that include supercoiling of topological domains reproduce several known features of interphase chromosomes
Source: Nucleic Acids Res. 2013 Dec 22;42(5):2848–55. doi: 10.1093/nar/gkt1353 (PMC3950722; doi:10.1093/nar/gkt1353)
Supplement: Supplementary Data [file supp_gkt1353_nar-03322-z-2013-File007.docx]

**Models that include supercoiling of topological domains reproduce several known features of interphase chromosomes**

Fabrizio Benedetti^a1^, Julien Dorier^a,b1^, Yannis Burnier^a,c1^ & Andrzej Stasiak^a^

^a^Center for Integrative Genomics, Faculty of Biology and Medicine, University of Lausanne, 1015-Lausanne, Switzerland.

^b^Vital-IT Group, SIB Swiss Institute of Bioinformatics, 1015-Lausanne, Switzerland.

^c^Laboratory of Particle Physics and Cosmology, Institute of Theoretical Physics, Ecole polytechnique fédérale de Lausanne EPFL, 1015-Lausanne, Switzerland.

^1^These authors contributed equally to this work.

Corresponding author:

Andrzej Stasiak, Center for Integrative Genomics, Faculty of Biology and Medicine, University of Lausanne, 1015-Lausanne, Switzerland. Phone: +4121 6924282, E-mail: [Andrzej.Stasiak@unil.ch](mailto:Andrzej.Stasiak@unil.ch)

Supplementary Data

Methods

Potentials.

To simulate thermally fluctuating chromatin fragments forming topological domains we employed HooMD-blue program (<http://codeblue.umich.edu/hoomd-blue>) using Brownian dynamics integration ([1](#_ENREF_1)). Topological domains were modelled as adequately constrained beaded chains composed of primary and secondary beads (see Fig. S1). Viscous drag of primary and secondary beads was set to 0.5 reduced units, the mass of primary beads was set to 1 and the mass of secondary beads to 0.1. The diameter of primary beads was set to 1 unit length. The mechanical properties of simulated beaded chains model were specified by four independent potentials:

1. Harmonic bond length potential, where the energy of a bond connecting two beads grows with the square of the displacement from the rest distance r0. Harmonic bond length potential is expressed by the formula V(r) = 0.5k(r-r0)^2^, where k = 2000ε0 and where ε0 is the reduced Lennard-Jones energy unit (<http://codeblue.umich.edu/hoomd-blue>). The r0 was set to 1 for bonds connecting two primary beads or two secondary beads. For bonds connecting axial secondary beads with the preceding primary beads the r0 was set to 0.5.
2. Harmonic bending potential that depends on the angle between two successive bonds and which grows with the square of the deflection angle from the preset rest angles α(0), β(0) and γ(0). The harmonic bending potential is expressed by the formulas:

$\begin{aligned} V\left( \alpha\right)=0.5\epsilon_{a}\left( \alpha_{i}-\alpha^{\left( 0 \right)} \right)^{2} \\ V\left( \beta\right)=0.5\epsilon_{b}\left( \beta_{i}-\beta^{\left( 0 \right)} \right)^{2} \\ V\left( \gamma\right)=0.5\epsilon_{g}\left( \gamma_{i}-\gamma^{\left( 0 \right)} \right)^{2} \end{aligned}$

where εa = 2ε0 and εb = εg = 500ε0. The rest bending angles α(0), β(0) and γ(0) were set to 0º, 180º and 90º, respectively (see Fig. S1).

1. Torsional potential that acts to diminish dihedral angles between sequential bonds joining axial and periaxial secondary beads (see Fig. S2) The potential is defined as

$V\left( \phi\right)=0.5k\left( 1-cos\left( \phi\right) \right)$

where k = 50ε0. The torsional potential guarantees that modelled molecules with a torsional stress can’t dissipate this stress by local swivelling. Instead, they form supercoils as this permits to decrease their originally entered torsional stress. The torsional potential is defined only for the bonds connecting axial and periaxial secondary beads (Fig. 1B), however a decrease of torsional stress between secondary beads drives writhing of entire chains used to model topological domains.

1. Excluded volume interactions between beads is expressed by cut Lennard-Jones potential:

$V\left( r \right)=4\epsilon\left[ \left( \sigma/r \right)^{12}+\left( \sigma/r \right)^{6}+\left( {127}/{16384} \right) \right]$

where ε= ε0 and σ = 1. This potential acts only between primary beads.

With respect to excluded volume interactions, secondary beads behave as phantom and can freely interpenetrate with primary and secondary beads. However, secondary beads can’t move freely with respect to other beads as their movement is constrained by bonds connecting them directly or indirectly to primary beads and these bonds are respecting bond length and specific bending potentials. Due to these bond potentials the equilibrium position of axial secondary beads is exactly in the middle between two consecutive primary beads. The bending potential of the bond connecting axial and periaxial secondary beads positions this bond orthogonally with respect to the bonds connecting two consecutive primary beads (see Fig. S1). The temperature for the system was set to 1 and the time step was set to 0.003 (reduced Lennard-Jones units).

Supercoiling.

In the absence of the torsional potential the periaxial secondary beads can freely rotate, independently from each other around the axis of the beaded chains. When the torsional potential is active all sequential pairs of bonds connecting axial and periaxial secondary beads tend to minimize their dihedral angle. In circular chains sequential pairs of bonds defining the dihedral angle form a closed circuit, which prevents torsional stress from dissipation by swivelling. Starting configurations of circular chains were created in such a way that the secondary periaxial beads follow a smooth line that is winding integer number of times around the circular chain formed by primary beads (See Fig. S3).

Construction of topological domains.

Individual topological domains were simulated as portions of circular chains (Fig. S2). The remaining portions of circular chains served only the accessory role of maintaining the torsional tension. The accessory subchains were not considered in the statistics of contacts. For systems composed of two or three joined circular chains (presented in Figs. 1-3) the accessory subchains were of the same length as the subchains representing modelled topological domains. For the system with 50 rings the insert-subchain was chosen to correspond to the average distance that would separate two border elements if they were in torsionally unconstrained linear polymer. That distance was calculated according to the Kratky-Porod equation:

$\sqrt{\left\langle R^{2} \right\rangle}=\left( {2L}_{p}^{2}\left( \left( \frac{s}{L_{p}} \right)-1+exp\left( \frac{s}{L_{p}} \right) \right) \right)^{1/2}$

To induce torsional tension into modelled circular chains we placed periaxial beads along a helical smooth line that was making an integral number of windings around the chain formed by the primary beads (Fig. S2). Subsequently, we switched on the torsional potential that minimizes the torsion angle between successive periaxial beads (see Fig. S1 and S2). In this setting the initial number of helical windings corresponds to the difference of the linking number between torsionally relaxed elastic ring and an elastic ring that before closure is turned a corresponding number of times. In an analogy with the terminology used to describe supercoiled DNA molecules we call this number of rotations as ΔLk ([2](#_ENREF_2)). As our simulated topological domains constituted only a part of circular molecules with a given ΔLk, we do not provide values of the total ΔLk per ring but normalize it to a stretch of 100 beads. The tested interval of ΔLk in our simulations ranged from 0 to 8 per 100 beads. To model a chromosomal region composed of three or two topological domains the rings that contained subchains representing modelled individual topological domains were connected with each other by short linkers containing four primary beads. In the case of the system with 50 rings the linkers ranged from 3 to 7 beads. The linkers were free to undergo axial rotation. During simulations the torsional potential drives the system to form supercoils since writhing permits to decrease the torsional stress (ΔTw) according to the formula

ΔLk = ΔTw + Wr

The ratio between torsional and bending modulus applied by us resulted in ca. 9:1 repartition of ΔLk into Wr and ΔTw.

Equilibration.

To find out whether our simulation runs were long enough to provide a meaningful estimation of thermal equilibrium properties, one needs to estimate the correlation time τ of the system and check whether the total simulation time is much larger than τ. The correlation time can be understood as the number of iterations needed to obtain a configuration not correlated with the initial configuration. A direct measure of τ is not possible, however a lower bound can be obtained by measuring the correlation time τA for a number of possible observables A and keeping the largest τA:

$\tau\geq\max_{a}\tau_{a}$

1. The correlation time τA of an observable A is usually estimated by measuring the autocorrelation function:

$C_{a}\left( n \right)=\frac{\left\langle A_{i+n}A_{i} \right\rangle-\left\langle A_{i+n} \right\rangle\left\langle A_{i} \right\rangle}{\left\langle A^{2} \right\rangle-\left\langle A \right\rangle^{2}}$

where < ... > denote thermal average.

2. Extracting τA by fitting

$C_{a}\left( n \right)\sim exp\left( {-n}/{\tau_{a}} \right)$

In general, the brownian dynamics evolution is described by more than one time scale, and the function CA(n) is better reproduced by a sum of exponentially decreasing function with different time scale τA,1, τA,2 ...

$C_{a}\left( n \right)=\sum_{k} B_{k}exp\left( {-n}/{\tau_{a,k}} \right)$

In this work, we decided to measure the correlation time of the radius of gyration τRg, which is a slowly evolving observable in this system. The corresponding autocorrelation functions are shown in figure S4. In our simulations, two correlation times τRg,1 and τRg,2 were needed to reproduce the autocorrelation function. These correlation times were obtained by fitting the autocorrelation function with

$C_{Rg}\left( n \right)=B_{1}exp\left( {-n}/{\tau_{Rg,1}} \right)+B_{2}exp\left( {-n}/{\tau_{Rg,2}} \right)$

Table S1 presents the measured τRg,2 along with the total number of iterations for each of the simulated models.


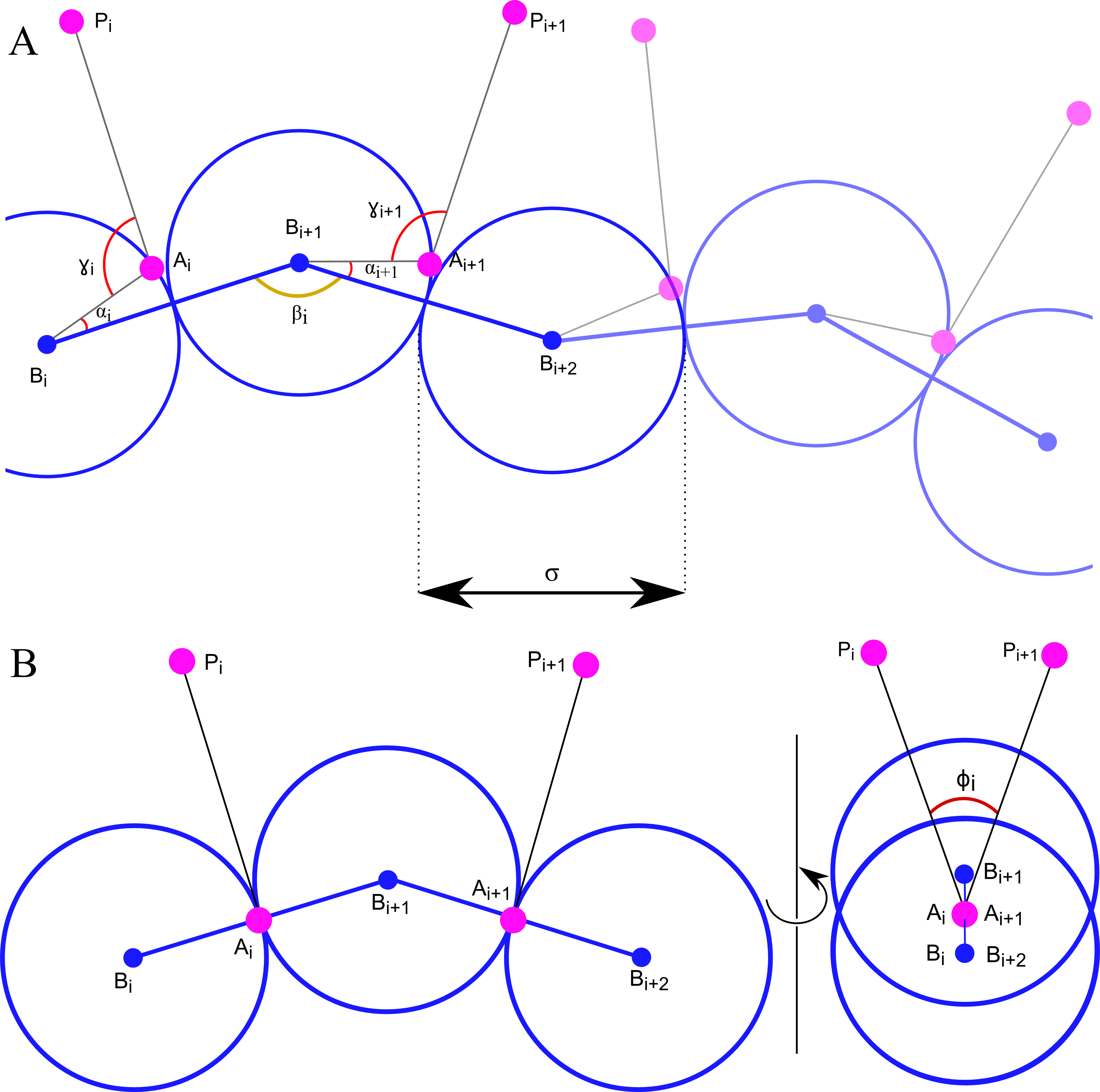


**Figure S1:** Connectivity and equilibrium bending angles of bonds constraining primary and secondary beads in modelled worm-like chains with bending and torsional resistance. Secondary beads (pink) are phantom with respect to excluded volume interactions with all other beads. Therefore, only primary beads (blue large circles) can be considered as representing elements of physical chains such as chromatin fibres. **A.** To provide insight into the connectivity and bending potentials the chain is presented in a configuration that is strongly distorted from its equilibrium configuration. The angles αi, βi and γi are constrained by the harmonic bending potential with the rest angles α^(0)^ = 0˚, β^(0)^ = 180˚ and γ^(0)^ = 90˚, respectively. **B.** Side and edge-on view of a short fragment of beaded chain shows how the dihedral angle between two sequential bonds connecting axial and periaxial secondary beads is measured. The angles φi are constrained by a torsional potential with rest angle φ(0) = 0˚, which tends to minimize the dihedral angle between sequential bonds joining axial and periaxial secondary beads.


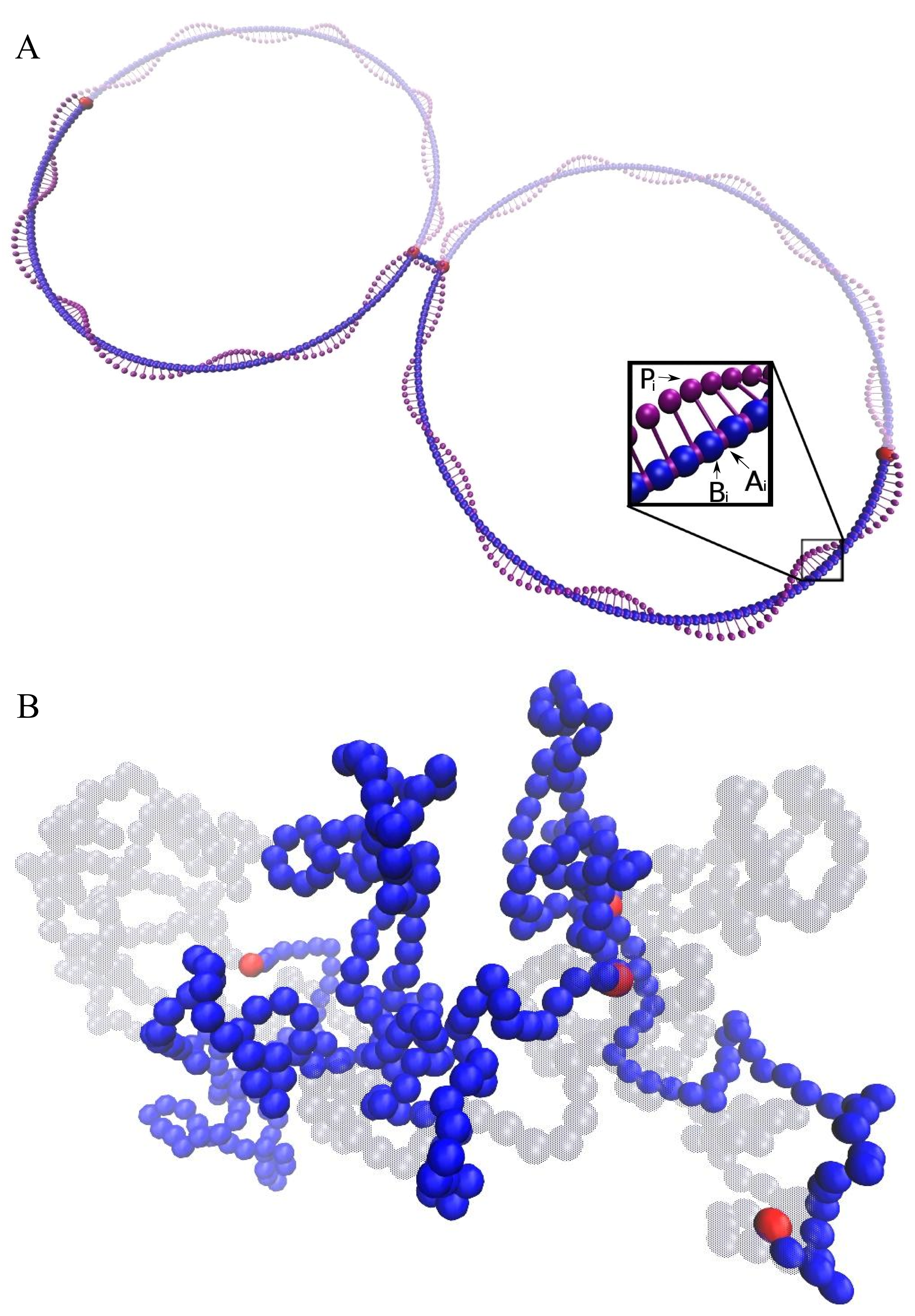


**Figure S2:** Starting (**A**) and equilibrated (**B**) configurations of modelled chromatin fragment composed of two topological domains. Intensively coloured chains are these that model topological domains, whereas lightly coloured chains form accessory linker chains that were not considered in the statistics of contacts. Red beads indicate borders of individual topological domains. **A.** The starting configuration to model two topological domains where each domain (composed of 100 beads) constitute a half of a circle. Notice that periaxial beads (Pi) follow a predetermined helical trajectory around the main chain but the torsional potential, minimizing the dihedral angle between all sequential bonds connecting axial and periaxial secondary beads is not yet switched on. In the shown example the ΔLk per 100 beads is 4. Once the simulation is started and all potentials are switched on, the torsional potential will act to decrease dihedral angles between sequential bonds connecting axial and periaxial secondary beads and this will drive formation of supercoils. **B.** A snapshot showing an equilibrated configuration of a modelled system with two topological domains (here one domain has 100 and another 200 beads). Intensively coloured beads belong to modelled topological domains that now show supercoiling. Lightly coloured beads belong to accessory chain which function is to maintain supercoiling while giving the end beads of topological domains a freedom to move independently from each other.


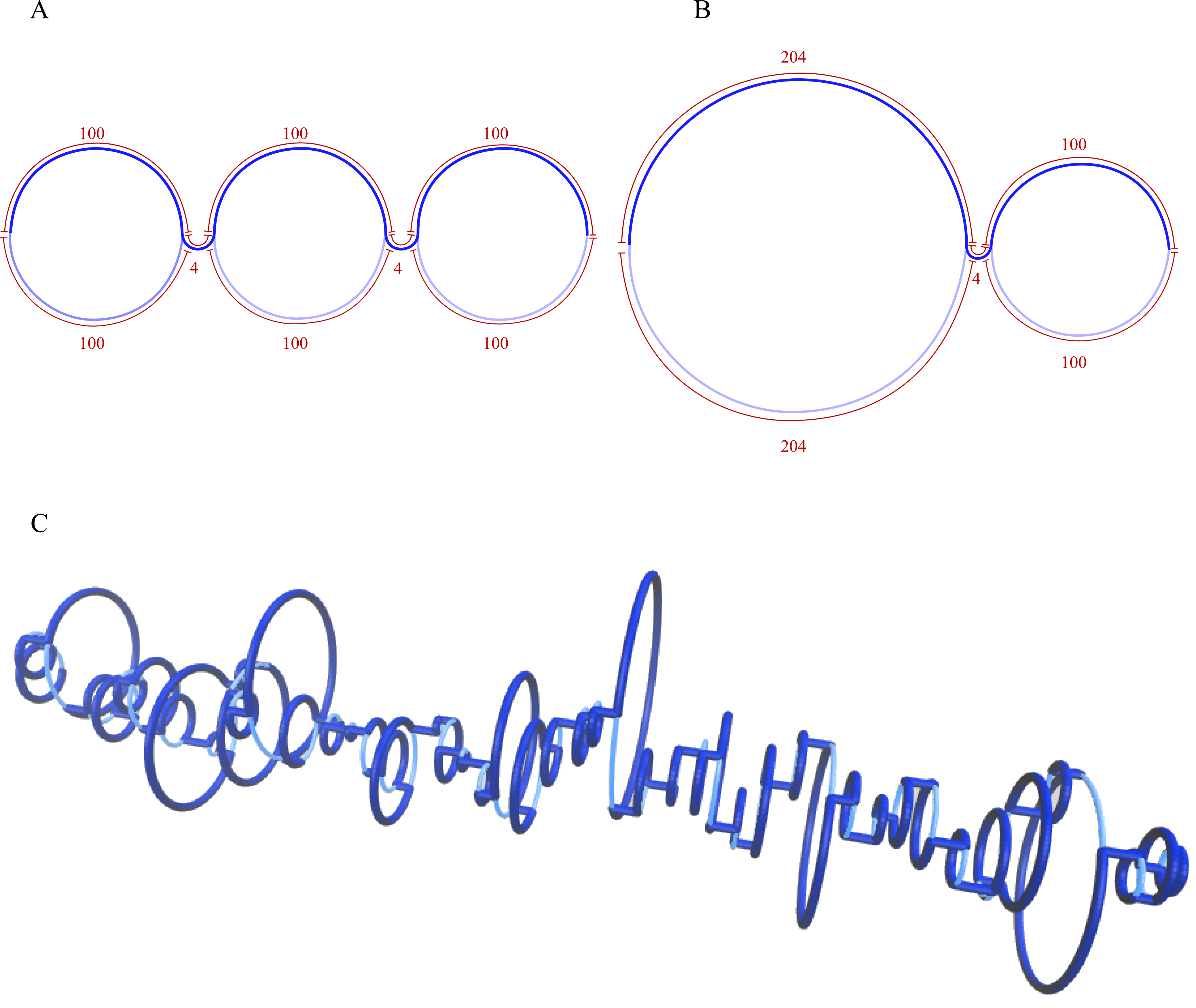


**Figure S3:** Schematic presentation of starting configurations of modelled chromosome fragments. **A** and **B**. Fragments composed of three and two topological domains were simulated assuming that each main chain bead in modelled topological domains represents 4000 bp. The chains representing modelled chromatin fragments are drawn with dark-blue line. Red lines show positions of insert subchains that were required to maintain supercoiling in individual topological domains but which were not entered into the statistics of contacts. The numbers of primary beads in individual topological domain, linkers and insert subchains are indicated. **C**. Starting configuration of chromosome fragment composed of 50 topological domains. The chain representing modelled chromosome fragment is shown as dark-blue thick cylinder whereas accessory insert subchains are shown as light blue thin cylinders.


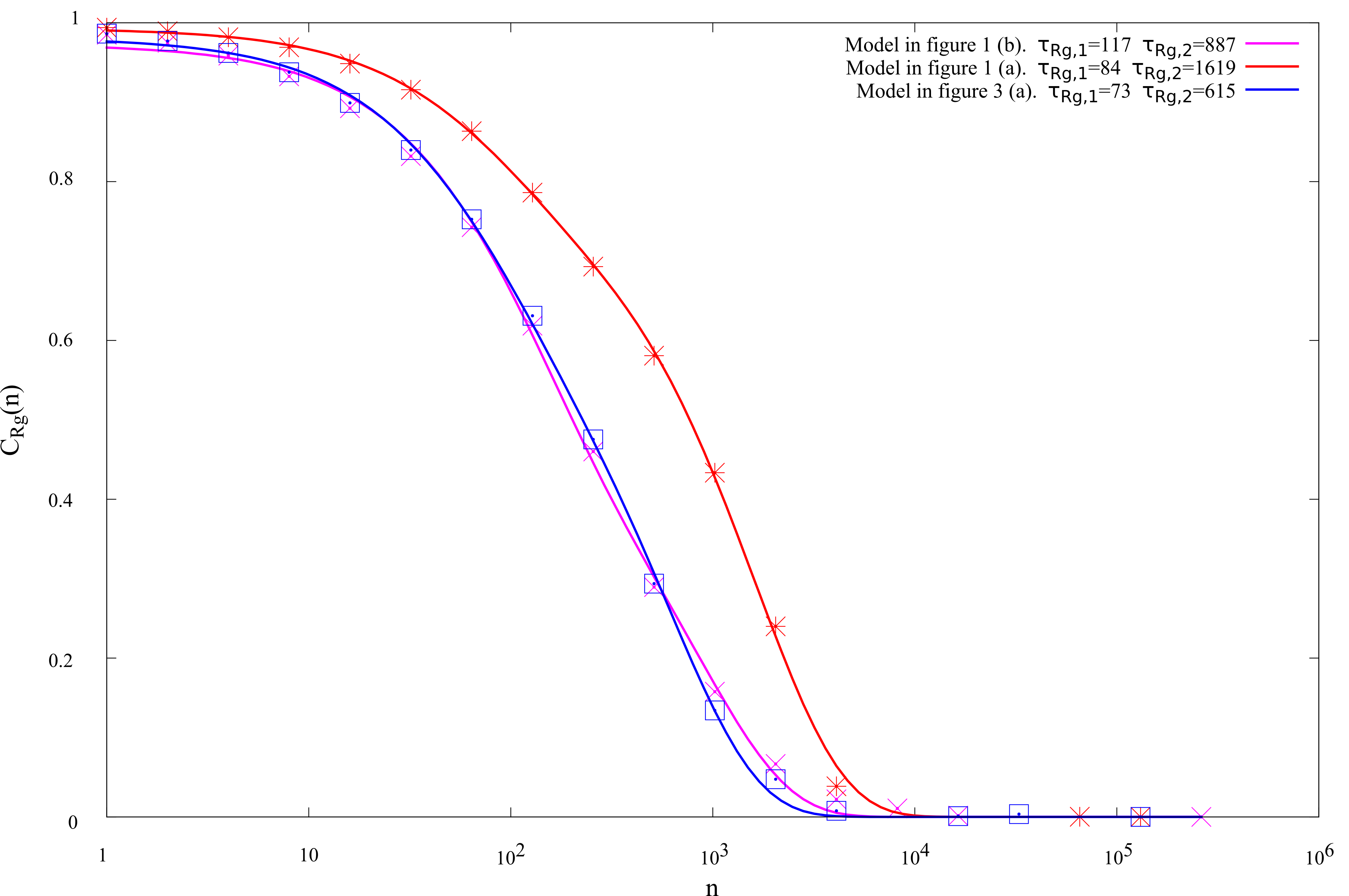


**Figure S4:** Autocorrelation of the radius of gyration for several simulated systems studied in this work. Data points and fits using double exponential decay function show how the correlation between evolving configurations decreases with the number of frames separating given two configurations. τRg;1 and τRg;2 are the correlation times, expressed in the number of separating frames, for the faster and slower mode of decorrelation of various systems. The slower mode is decisive for the determination of the correlation time of the given system.


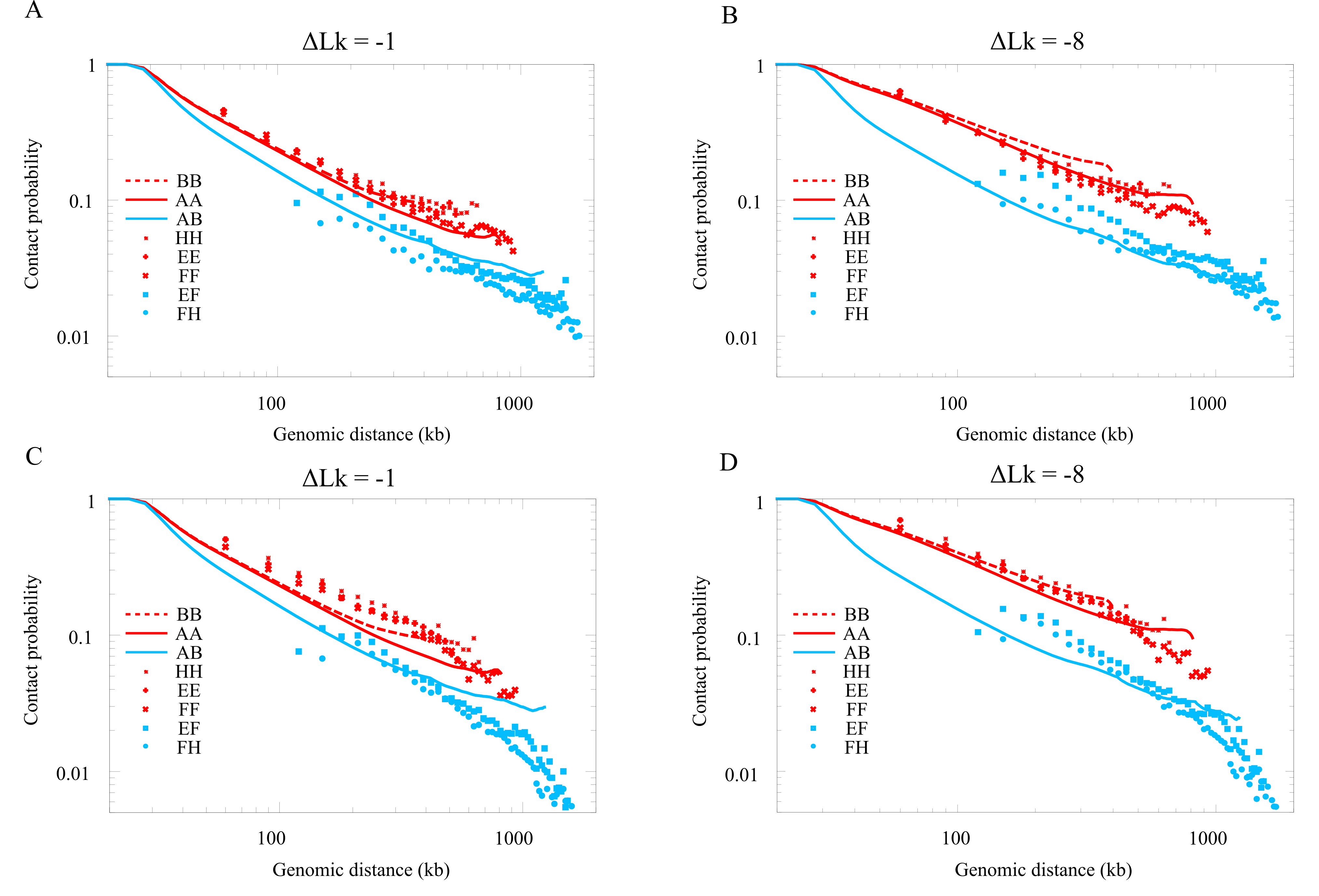


**Figure S5:** Comparison of simulations with experimental data. Average contact probability profiles obtained in simulations of chromatin fibres composed of two weakly (**A** and **C**) or strongly supercoiled topological domains (**B** and **D**) are compared with experimental 3C data. Notations AA, BB and AB indicate intra- and inter-domain contacts, respectively, for simulated topological domains presented in Fig. 1. The experimental 3C data points (shown as scatter plots) correspond to contacts within and between topological domains E, F and H in the mouse X-chromosome inactivation centre studied by Nora et al. ([3](#_ENREF_3)). Data in **A** and **B** are for differentiated mouse embryonic fibroblasts, whereas data in **C** and **D** are for mutant mouse embryonic stem cell line that is defective in H3K27me3 histone methylation (see SI and deposited data accompanying publication by Nora et al. ([3](#_ENREF_3))).


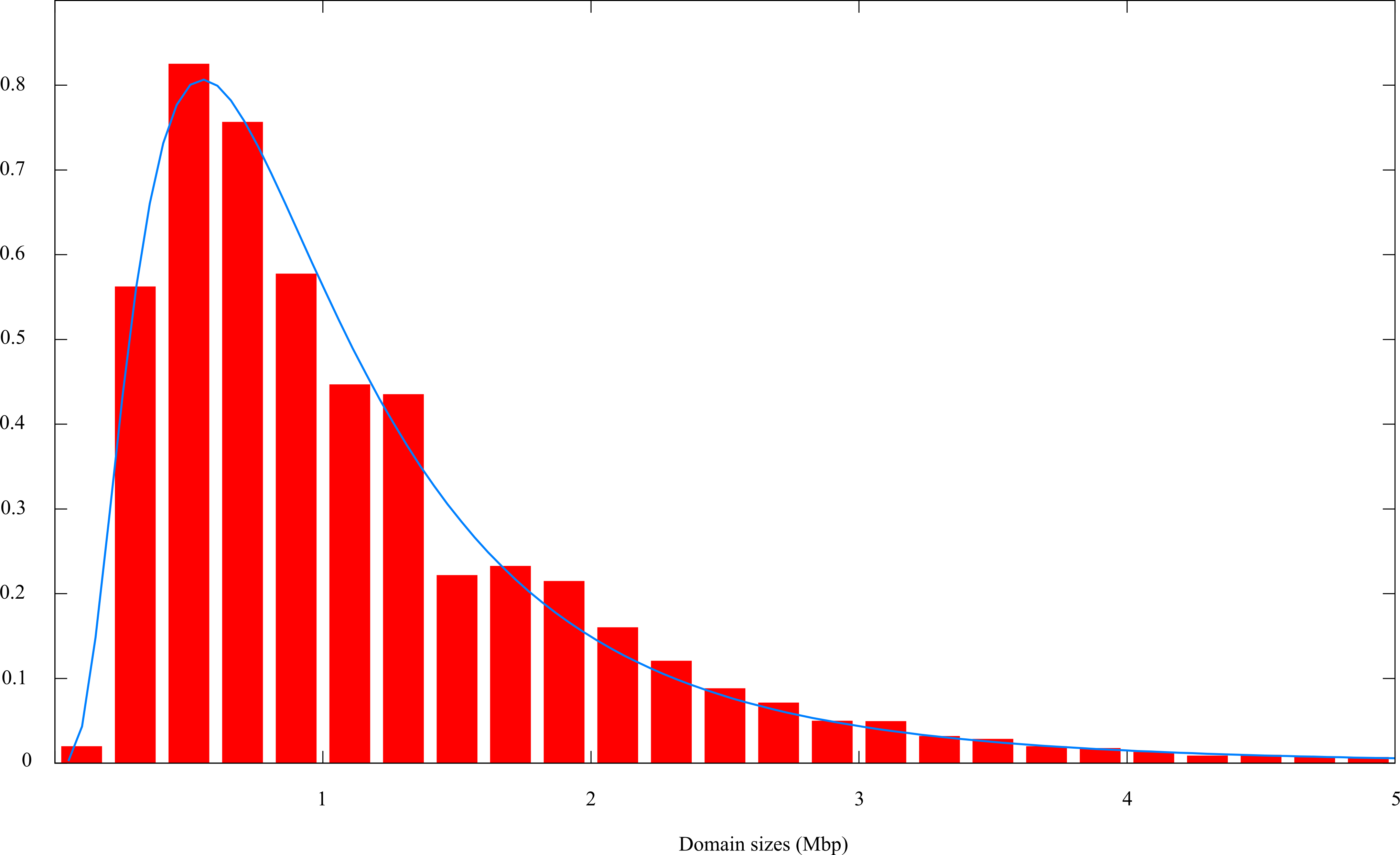


**Figure S6:** Experimentally observed probability distribution of sizes of topological domains. The distribution is obtained from the data deposited by Dixon et al. ([4](#_ENREF_4)), which were obtained after analysis of mouse embryonic stem cells, human embryonic stem cells, human IMR90 fibroblasts and cells from mouse cortex (see <http://chromosome.sdsc.edu/mouse/hi-c/download.html>).

**Table S1**

| System presented in | Frames # | τ1 | τ2 | # of correlation times |
| --- | --- | --- | --- | --- |
| Figure 1(a) | 316798 | 117 | 887 | 357 |
| Figure 1(b) | 556888 | 84 | 1619 | 344 |
| Figure 3(a) | 498300 | 73 | 615 | 810 |

**Table S1:** Frames, τ1, τ2 and number of correlation times (i.e. Frames #/(max )) for the systems studied.

References:

1. Anderson, J.A., Lorenz, C.D. and Travesset, A. (2008) Micellar crystals in solution from molecular dynamics simulations. *J Chem Phys*, **128**, 184906.

2. Bates, A.D. and Maxwell, A. (2005) *DNA Topology*. Oxford University Press, Oxford.

3. Nora, E.P., Lajoie, B.R., Schulz, E.G., Giorgetti, L., Okamoto, I., Servant, N., Piolot, T., van Berkum, N.L., Meisig, J., Sedat, J. *et al.* (2012) Spatial partitioning of the regulatory landscape of the X-inactivation centre. *Nature*, **485**, 381-385.

4. Dixon, J.R., Selvaraj, S., Yue, F., Kim, A., Li, Y., Shen, Y., Hu, M., Liu, J.S. and Ren, B. (2012) Topological domains in mammalian genomes identified by analysis of chromatin interactions. *Nature*, **485**, 376-380.
